# Supplementary material for: Inhibition of Amyloid Beta Aggregation and Deposition of Cistanche tubulosa Aqueous Extract
Source: Molecules. 2019 Feb 14;24(4):687. doi: 10.3390/molecules24040687 (PMC6412839; doi:10.3390/molecules24040687)
Supplement: Supplementary file 1 [file molecules-24-00687-s001.pdf]

Supporting Information

Inhibition of amyloid beta aggregation and deposition of *Cistanche tubulosa* aqueous extract

Chien-Liang Chao [1], Hsin-Wen Huang [1], Hui-Chi Huang [2], Hsin-Fan Chao [1], Shuen-Wen Yu [1], Muh-Hwan Su [1,3], Chao-Jih Wang [1,4]* and Hang-Ching Lin [1,3]*

[1] Sinphar Pharmaceutical Co., Ltd., Sinphar group, Yilan 269, Taiwan; chaokmc@gmail.com (C.-L.C.); lobar@sinphar.com.tw (H.-W.H); hfchao@sinphar.com.tw (H.-F.C); ysw@sinphar.com.tw (S.-W.Y); smh1027@sinphar.com.tw (M.-H.S); hcl@sinphar.com.tw (H.-C.L)

[2] Department of Chinese Pharmaceutical Sciences and Chinese Medicine Resources, China Medical University, Taichung 404, Taiwan; hchuang@mail.cmu.edu.tw

[3] School of Pharmacy, National Defense Medical Center, Taipei 114, Taiwan; smh1027@sinphar.com.tw (M.-H.S); hcl@sinphar.com.tw (H.-C.L)

[4] Sinphar Tian-Li Pharmaceutical Co., Ltd., Sinphar group, Hangzhou 311100, China; zrwang@sinphar.com.tw

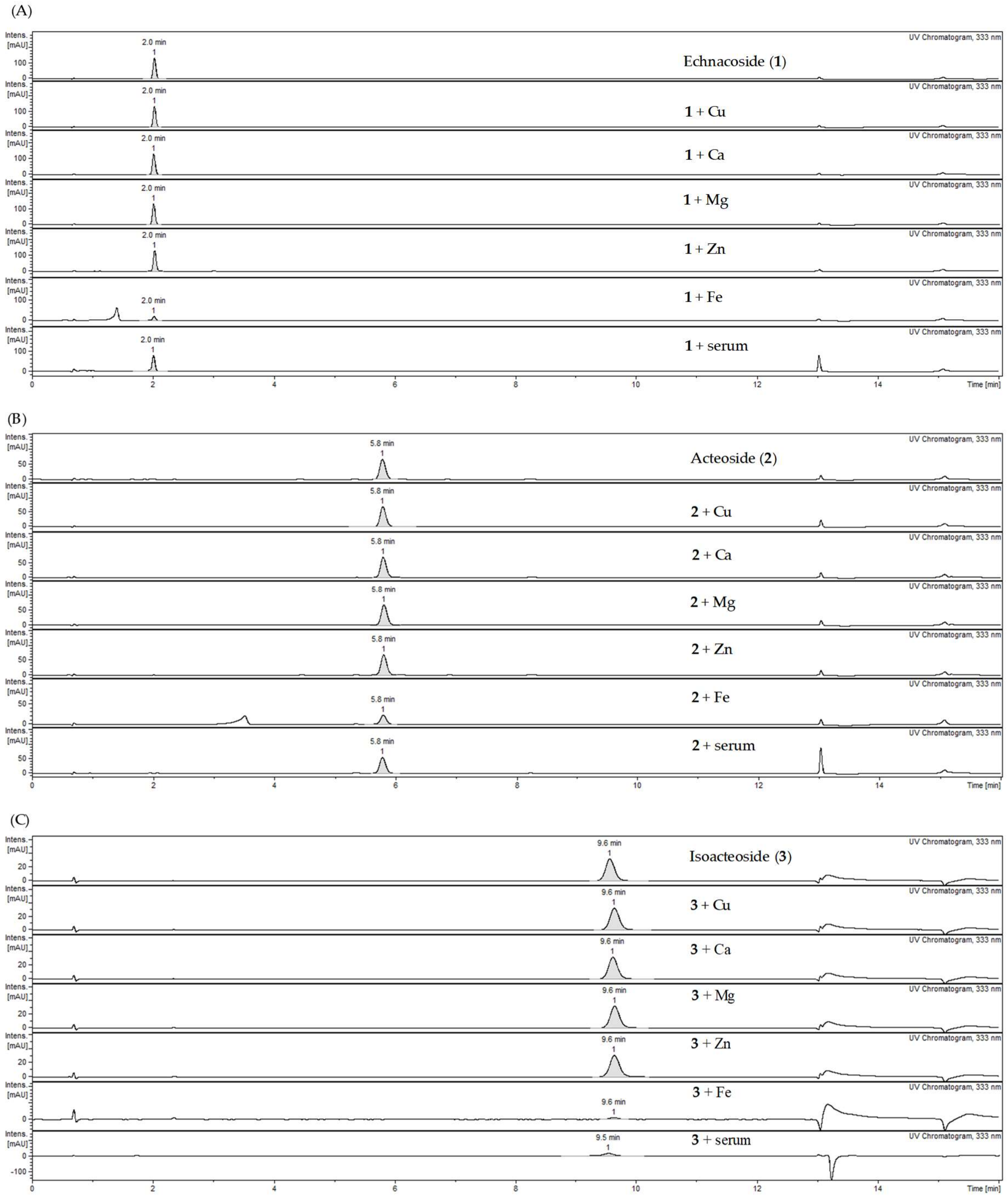

Figure 1S. Measure of chelation activity retained for the phenylethanoid glycosides, (a) compound **1**, (b) compound **2** and (c) compound **3** in the presence of copper (Cu), calcium (Ca), magnesium (Mg), zinc (Zn), iron (Fe), and rat serum by UPLC analysis. The data represented mean ± S.D., n=3. ***p<0.001.

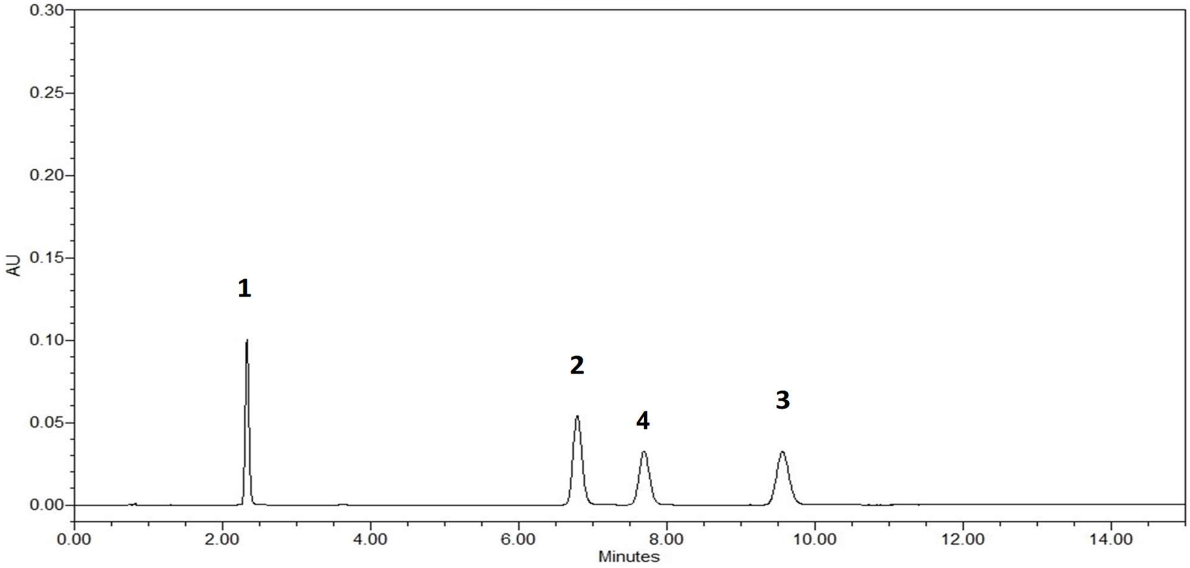

Figure 2S. The UPLC chromatogram of CTE. Echinacoside (**1**), acteoside (**2**), isoacteoside (**3**), and tubuloside A (**4)** were the components of CTE.

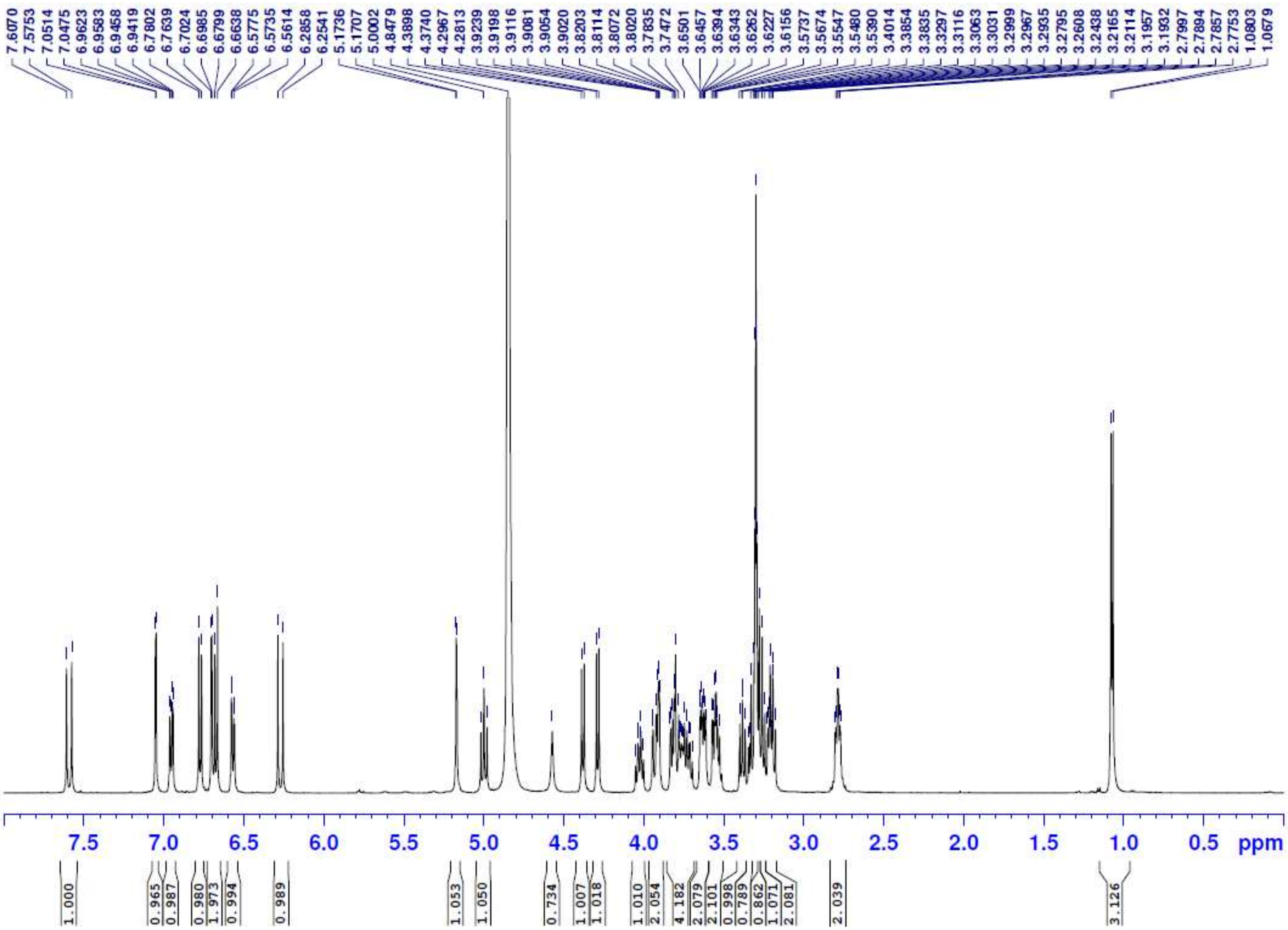

Figure 3S. The ^{1}H NMR (500 MHz, $CDCl_3$) data of **1**

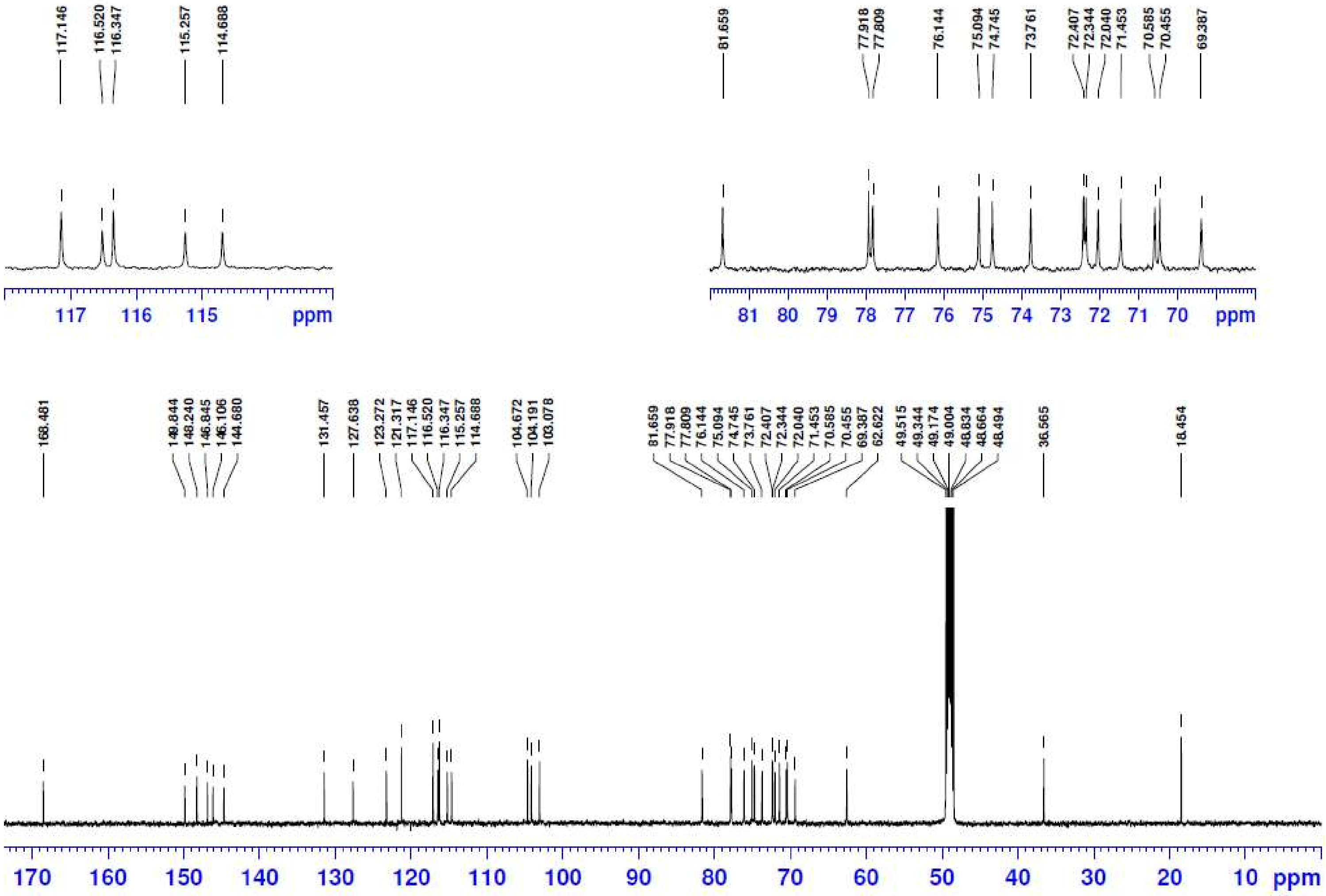

Figure 4S. The ^{13}C NMR (125 MHz, $CDCl_3$) data of **1**

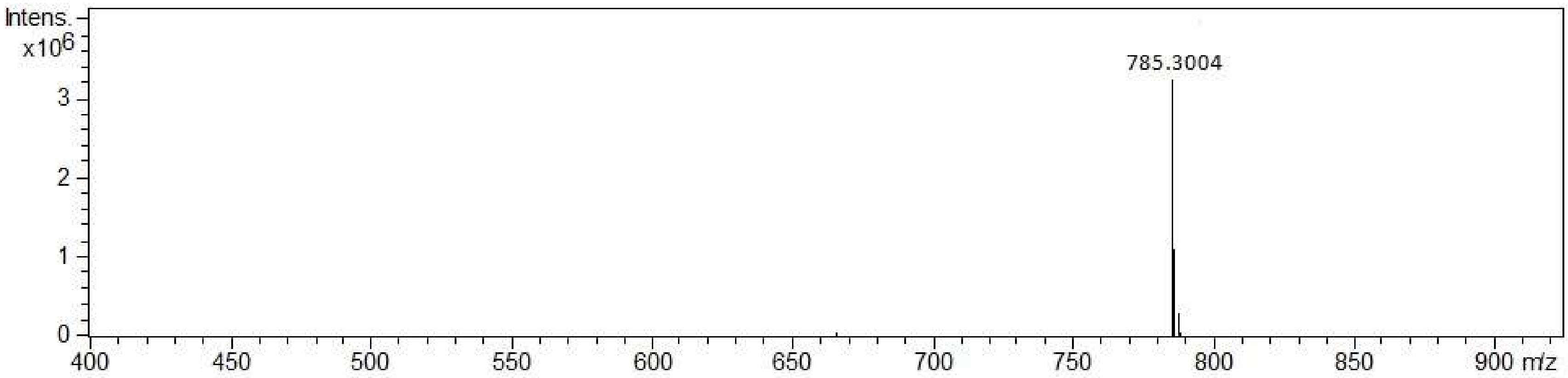

Figure 5S. The ESI-MS spectrum of **1**

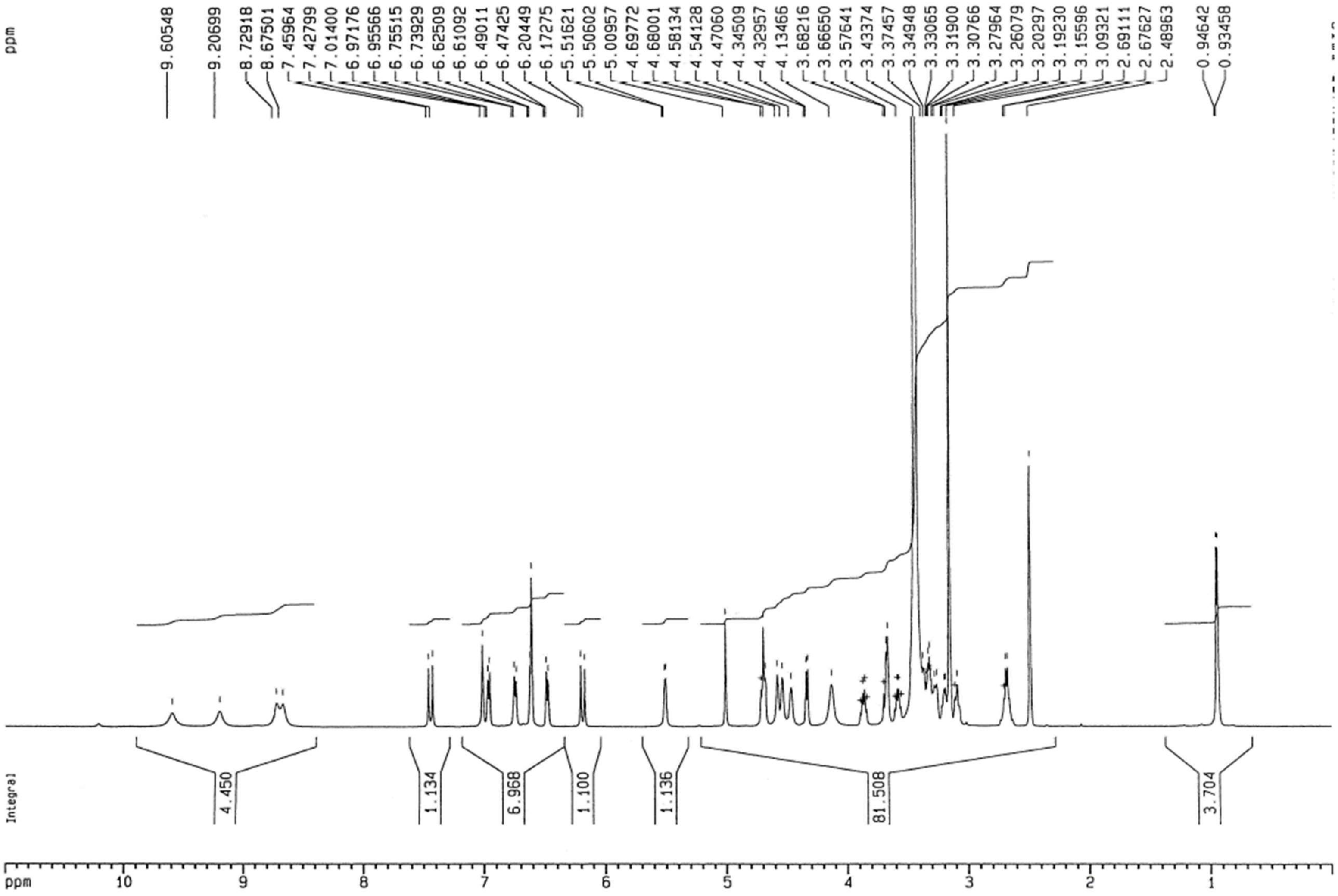

Figure 6S. The ^{1}H NMR (500 MHz, DMSO-d6) data of **2**

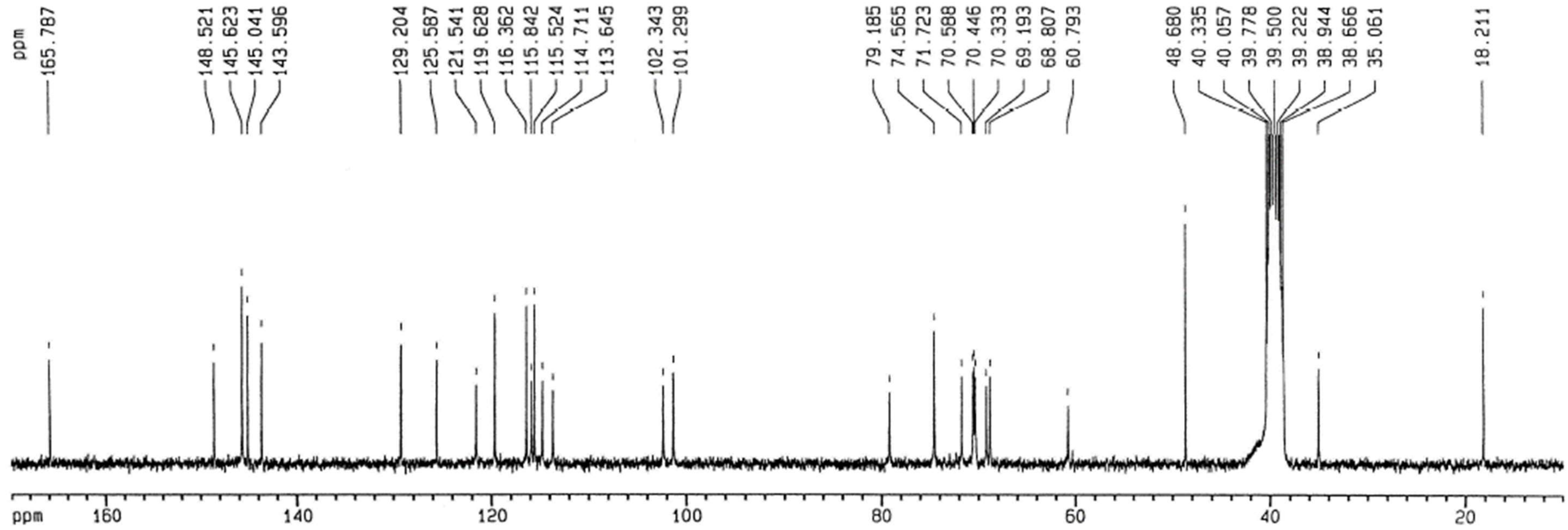

Figure 7S. The ^{13}C NMR (125 MHz, DMSO-d_6) data of **2**

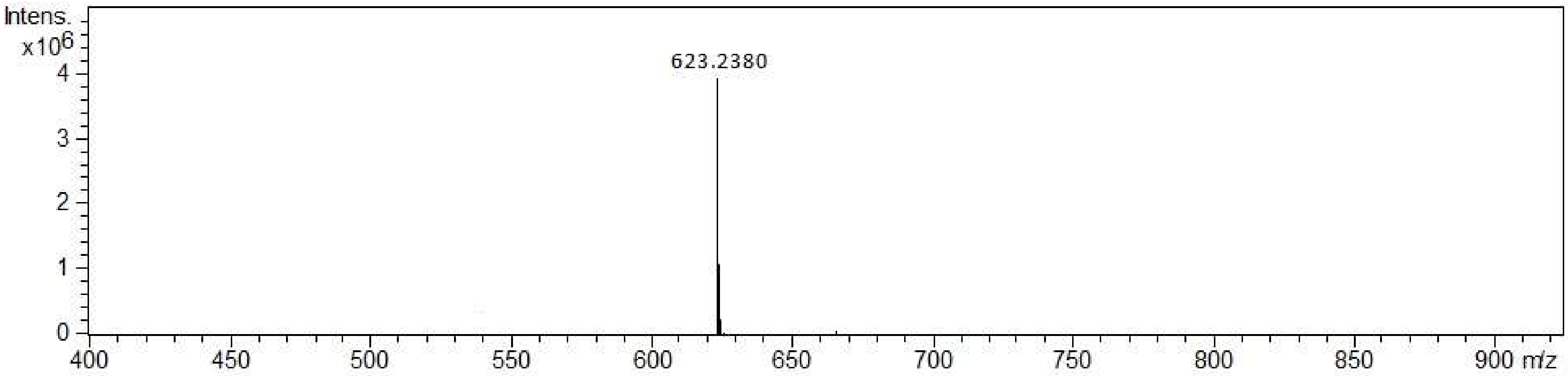

Figure 8S. The ESI-MS spectrum of **2**

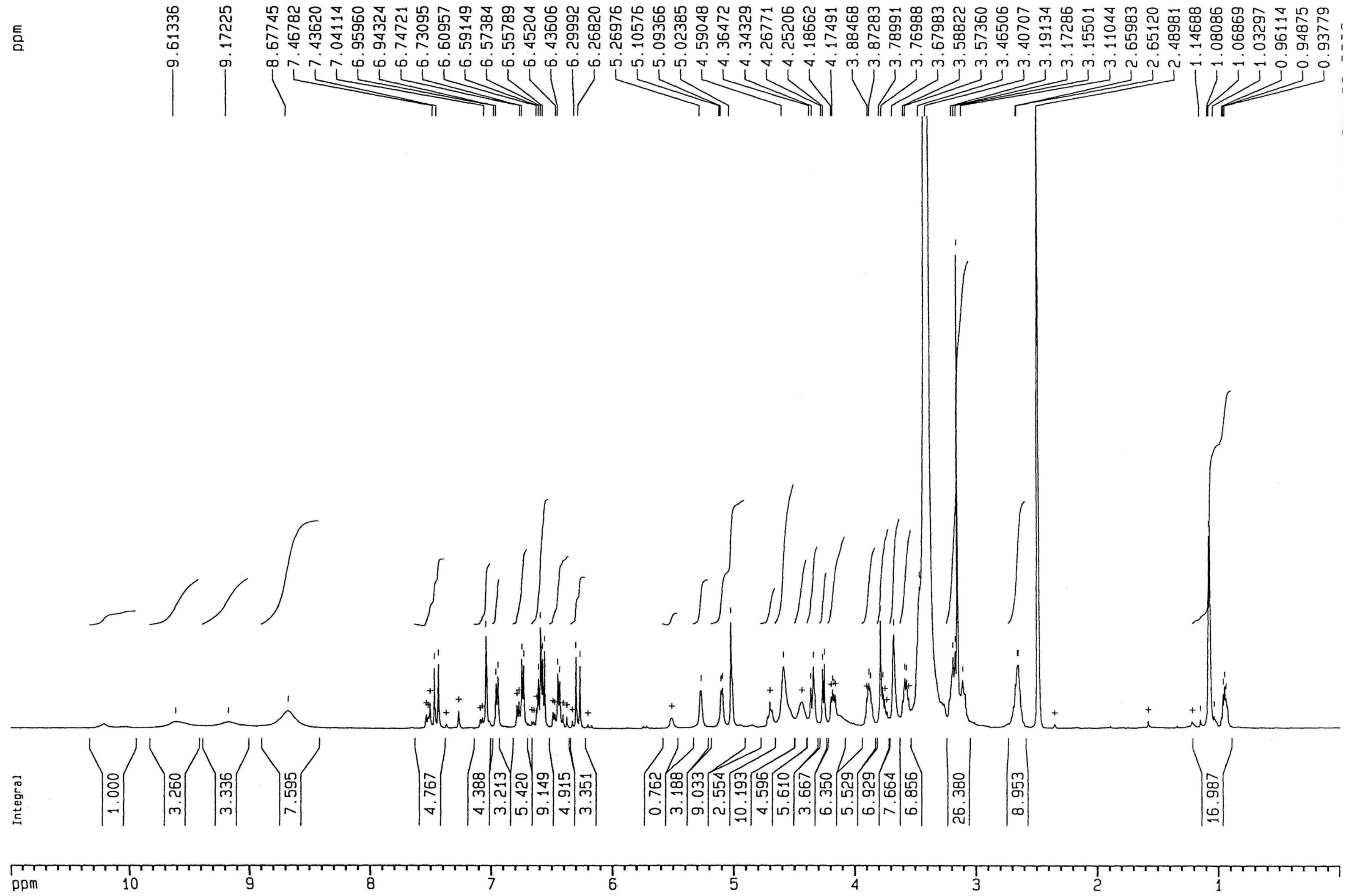

Figure 9S. The ^{1}H NMR (500 MHz, DMSO-d_6) data of **3**

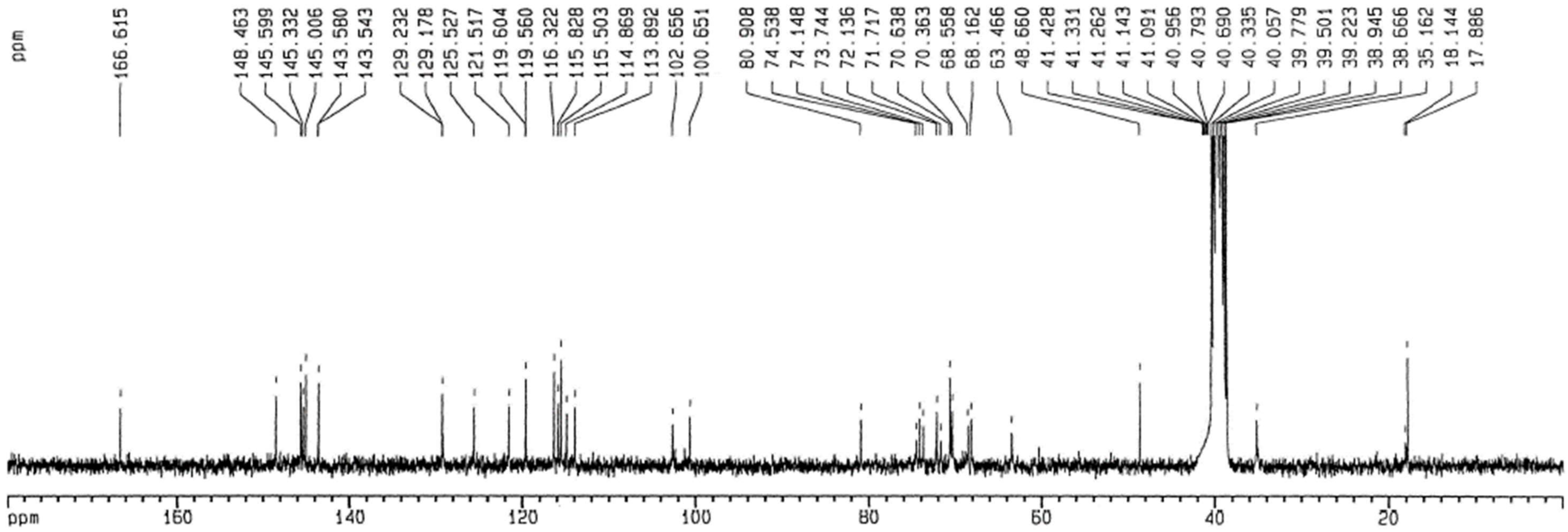

Figure 10S. The ^{13}C NMR (125 MHz, DMSO-d_6 data of **3**

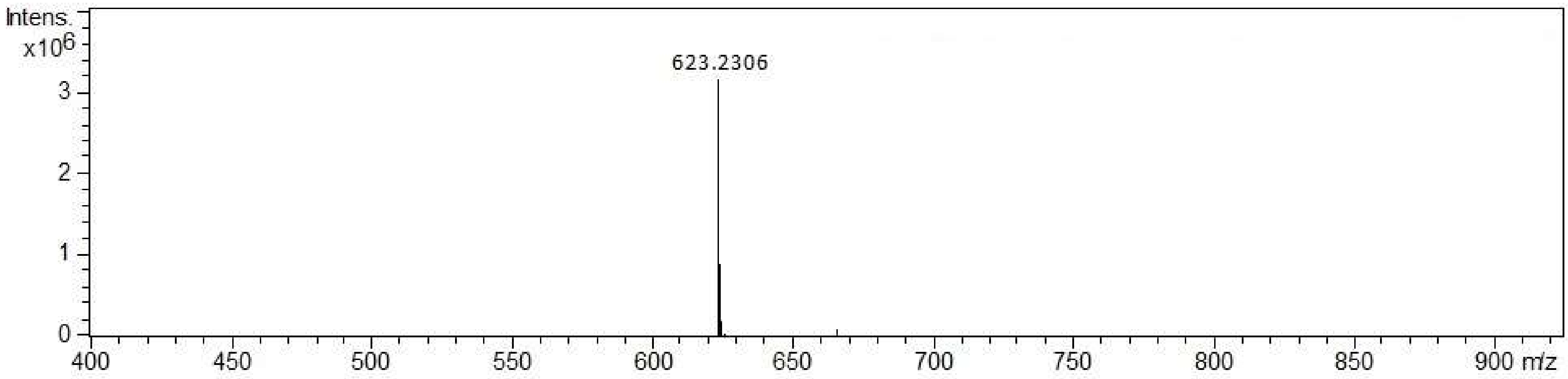

Figure 11S. The ESI-MS spectrum of **3**.
